# A pilot study of participatory and rapid implementation approaches to increase depression screening in primary care

**DOI:** 10.1186/s12875-021-01550-5

**Published:** 2021-11-16

**Authors:** Briana S. Last, Alison M. Buttenheim, Anne C. Futterer, Cecilia Livesey, Jeffrey Jaeger, Rebecca E. Stewart, Megan Reilly, Matthew J. Press, Maryanne Peifer, Courtney Benjamin Wolk, Rinad S. Beidas

**Affiliations:** 1 Department of Psychology, University of Pennsylvania, Philadelphia, PA USA; 2 Department of Family and Community Health, School of Nursing, University of Pennsylvania, Philadelphia, PA USA; 3 Center for Health Incentives and Behavioral Economics (CHIBE), University of Pennsylvania, Philadelphia, PA USA; 4 Leonard Davis Institute of Health Economics, University of Pennsylvania, Philadelphia, PA USA; 5 Department of Psychiatry, Perelman School of Medicine, University of Pennsylvania, Philadelphia, PA USA; 6 Department of Medicine, University of Pennsylvania Perelman School of Medicine, Philadelphia, PA USA; 7 Primary Care Service Line, University of Pennsylvania Perelman School of Medicine, Philadelphia, PA USA; 8 Department of Family Medicine and Community Health, University of Pennsylvania Perelman School of Medicine, Philadelphia, PA USA; 9 Penn Implementation Science Center at the Leonard Davis Institute of Health Economics (PISCE@LDI), University of Pennsylvania, Philadelphia, PA USA; 10 Department of Medical Ethics and Health Policy, University of Pennsylvania Perelman School of Medicine, Philadelphia, PA USA; 11 Penn Medicine Nudge Unit, University of Pennsylvania Health System, Philadelphia, PA USA

**Keywords:** Depression screening, Primary care, Participatory research, Rapid implementation, Implementation strategy design

## Abstract

**Background:**

Most individuals with depression go unidentified and untreated. In 2016 the US Preventive Services Task Force released guidelines recommending universal screening in primary care to identify patients with depression and to link them to treatment. Feasible, acceptable, and effective strategies to implement these guidelines are needed.

**Methods:**

This three-phased study employed rapid participatory methods to design and test strategies to increase depression screening at Penn Medicine, a large health system with 90 primary care practices. First, researchers solicited ideas and barriers from stakeholders to increase screening using an innovation tournament—a crowdsourcing method that invites stakeholders to submit ideas to address a workplace challenge. Second, a panel of stakeholders and scientists deliberated over and ranked the tournament ideas. An instant runoff election was held to select the winning idea. Third, the research team piloted the winning idea in a primary care practice using rapid prototyping, an approach that quickly refines and iterates strategy designs.

**Results:**

The innovation tournament yielded 31 ideas and 32 barriers from diverse stakeholders (12 primary care physicians, 10 medical assistants, 4 nurse practitioners, 2 practice managers, and 4 patient support assistants). A panel of 6 stakeholders and scientists deliberated on the ideas and voted for patient self-report (i.e., through tablet computers, text message, or an online patient portal) as the winning idea. The research team rapid prototyped tablets in one primary care practice with one physician over 5 five-hour shifts to examine the feasibility, acceptability, and effectiveness of the strategy. Most patients, the physician, and medical assistants found the tablets acceptable and feasible. However, patient support assistants struggled to incorporate them in their workflow and expressed concerns about scaling up the process. Depression screening rates were higher using tablets compared to usual care; follow-up was comparable between tablets and usual care.

**Conclusions:**

Rapid participatory methods engaged and amplified the voices of diverse stakeholders in primary care. These methods helped design an acceptable and feasible implementation strategy that showed promise for increasing depression screening in a primary care setting. The next step is to evaluate the strategy in a randomized controlled trial across primary care practices.

**Supplementary Information:**

The online version contains supplementary material available at 10.1186/s12875-021-01550-5.

Depression is a leading cause of disability, affecting between 8 and 17% of the population [1–6]. Untreated depression is associated with significant medical comorbidity, functional impairment, low medication adherence, and increased risk of mortality [2, 7, 8]. Though a variety of mental health interventions reduce symptoms and other sequelae, only a fraction of people with depression receive treatment [9–12]. One major challenge is the identification of individuals with depression [13, 14]; as many as half of cases of depression go undiagnosed [15, 16].

Primary care is an optimal place to identify individuals with depression. First, due to the association between psychiatric disorders and physical diseases, primary care practices serve individuals with elevated rates of depression compared to the general population [17–21]. Second, patients overwhelmingly trust their primary care clinicians and may be more willing to seek treatment with their encouragement [22, 23]. Screening and subsequently treating patients for depression in primary care settings is effective at increasing response to treatment and remission, controlling physical disease, and reducing total healthcare costs [24–27].

In response to this mounting evidence, national organizations, payers, policymakers, and health systems have begun to transform their depression screening practices. The US Preventive Services Task Force now recommends that health systems implement universal depression screening protocols [28, 29]. These recommendations prompted the Centers for Medicare & Medicaid Services (CMS) to cover annual depression screening for Medicare beneficiaries in primary care and to financially incentivize health system universal screening practices [30]. The National Committee for Quality Assurance, the national accrediting body that approves quality of care performance metrics, approved the Patient Health Questionnaires (PHQ) as potential depression screeners. These include the PHQ-2, a well-validated 2-item tool shown to be sensitive and specific to Major Depressive Disorder, and the PHQ-9, the 9-item version of the questionnaire [31]. The PHQ-2 and PHQ-9 have become the depression assessments of choice for many health systems.

Despite payer incentives, there have been few specific guidelines to support health system implementation of universal depression screening in primary care. Many health systems have attempted to increase depression screening and have encountered several barriers [32]. Just before the 2016 universal screening guidelines were implemented, nationally representative studies found that depression screening occurred at rates of just 3-4% in primary care practices [33, 34]. Thus, acceptable and feasible implementation strategies to increase depression screening in primary care are needed.

In our study, we used participatory and rapid implementation methods that involved stakeholders across primary care at the University of Pennsylvania Health System (Penn Medicine) to design and pilot strategies to increase depression screening and follow-up. Stakeholder participation ensures the acceptability and feasibility of implementation efforts [35]. Large-scale health system initiatives to increase universal depression screening must incorporate the interests of all stakeholders involved in the process: leaders from Primary Care and Psychiatry, clinicians, staff, and patients. In addition to growing recognition that stakeholder input is crucial to implementation success, it is now acknowledged that the gap between research and practice is sometimes prolonged by traditional randomized controlled trial implementation studies, that, though rigorous, are resource intensive. Rapid implementation methods are an increasingly popular approach to design strategies [36–39]. Without sacrificing the systematicity of more rigorous approaches, rapid implementation methods accelerate the pace of data collection to immediately identify problems in system changes and are therefore designed to pilot and fine-tune implementation strategies. Rapid implementation allows health systems engaged in quality improvement projects and researchers interested in developing generalizable implementation strategies to “fail fast” and quickly refine their strategies before they scale up their approach. In other words, rapid implementation approaches are flexible methods that can be used by both health systems seeking to generate context-specific knowledge for quality improvement and by researchers seeking to design strategies that can be tested in other health service contexts. Our study provides health systems with a specific strategy to improve depression screening and assessment in primary care settings, as well as a general framework and set of methods to design implementation strategies.

## General methods

### Study context

In 2018, Penn Medicine’s Department of Psychiatry and Primary Care Service Line initiated a staged and adapted implementation of the evidence-based Collaborative Care Model—referred to as the Penn Integrated Care program (PIC) [40]—an approach for managing psychiatric disorders in primary care that combines mental and physical health services into a single setting [41, 42]. Healthcare professionals from multiple disciplines work together to coordinate care and treat patients. The Collaborative Care Model improves access to care and has shown promise in improving clinical outcomes for psychiatric and physical health conditions [43, 44].

Penn Medicine launched the PIC program in eight of its 90 primary care practices. These eight practices in Philadelphia serve over 90,000 patients and range in size from 4 to 26 primary care clinicians. The PIC program is modeled on the traditional Collaborative Care Model, though it also includes a “Resource Center” to assess and triage Penn Medicine primary care patients in need of any mental health services. We decided to conduct this study in PIC practices based on consistent feedback from primary care stakeholders that depression screening can only be ethically conducted if follow-up care is readily available.

When we began our study in February 2019, across all 90 of Penn Medicine’s primary care practices (including its PIC practices), depression screening was variably implemented. Around 40% of eligible patients were screened for depression annually, with eligibility defined by CMS as all adult patients with a primary care office visit who are not already diagnosed with a mood disorder [45]. The PHQ-2 was typically administered verbally by a medical assistant (MA) before the patient saw their primary care clinician as their vital signs were checked (i.e., temperature, blood pressure, etc.). Depending on the practice, the PHQ-2 was not always entered in the same location in the patient’s electronic health record, Epic©. If the patient screened positive on the PHQ-2 (a score of > 2), the MA would provide a paper-and-pencil version of the PHQ-9 for the patient to complete and hand-off to their primary care clinician when they entered the exam room. The primary care clinician would then be expected to manually enter the patient’s PHQ-9 scores in Epic© and follow-up if clinically indicated.

### Overview of participants and procedures

Our study includes three distinct phases, all of which were conducted in PIC practices. In Phase 1, we conducted an innovation tournament to generate strategies to improve depression screening. Innovation tournaments are a novel participatory method in which stakeholders are invited to submit their ideas to address a specific challenge faced by a workplace, industry, or service system [46, 47]. We targeted leaders from Primary Care and Psychiatry, clinicians, and staff involved in depression screening (i.e., who interface with the PHQ-2 and PHQ-9) as our key stakeholders. We analyzed responses from the innovation tournament using a content coding approach to organize the ideas into themes. In Phase 2, we held a panel with expert stakeholders and scientists to discuss the ideas. After extended deliberation, the panel voted on a winning idea from the tournament. In Phase 3, we piloted the winning idea in a PIC practice with one physician over 5 five-hour shifts. When piloting, we used a mixed methods rapid implementation approach called rapid prototyping, which systematically tested and refined the strategy to ensure the implementation method was acceptable and feasible [38, 39, 48, 49]. All study procedures were approved by the University of Pennsylvania’s Institutional Review Board.

### Phase 1 — the innovation tournament

#### Methods

##### Procedure

In order to generate acceptable and feasible methods to increase depression screening in primary care, we deployed an innovation tournament. Innovation tournaments are designed to “democratize innovation” by increasing participation and engagement among stakeholders that have first-hand experience with a problem but are not typically consulted in workplace transformations [47]. Cash prizes incentivize tournament participation and creative idea generation [50]. Innovation tournaments have been shown to be effective in Penn Medicine and community settings for designing implementation strategies that empower stakeholders and increase investment in the suggested ideas [47, 51]. The innovation tournament was based on methods developed by the Penn Medicine Center for Health Care Innovation and prior work with clinicians [47, 51, 52].

##### Tournament platform

The Penn Medicine Center for Health Care Innovation hosts a web-based platform called “Your Big Idea” to run tournaments. Researchers post prompts about a healthcare challenge to crowdsource solutions. Participants can respond to these prompts, called “Idea Challenges,” with an idea, rate other participants’ ideas on a 1–5 “star” rating scale, and comment on other participants’ ideas.

##### Tournament prompts

The Idea Challenges were designed through a participatory process. The research team developed several different prompts that asked participants how they would improve depression screening. After consultation with leaders in the Penn Medicine Center for Health Care Innovation with experience conducting innovation tournaments, Idea Challenges were narrowed down to three options and a question was added about barriers to depression screening. The research team attended staff meetings (attended by practice leaders, clinicians, staff, and administrators) at two of the PIC practices and asked attendees to respond to each of the three Idea Challenges. Meeting attendees discussed the advantages and disadvantages of each prompt. At both meetings, the consensus was to focus the question on increasing screening rates. The final prompt was: “What’s your big idea for increasing depression screening rates in the primary care setting?” The barrier question was “What currently gets in the way of screening patients for depression in your clinic?”

##### Recruitment

**Fig. 1:**
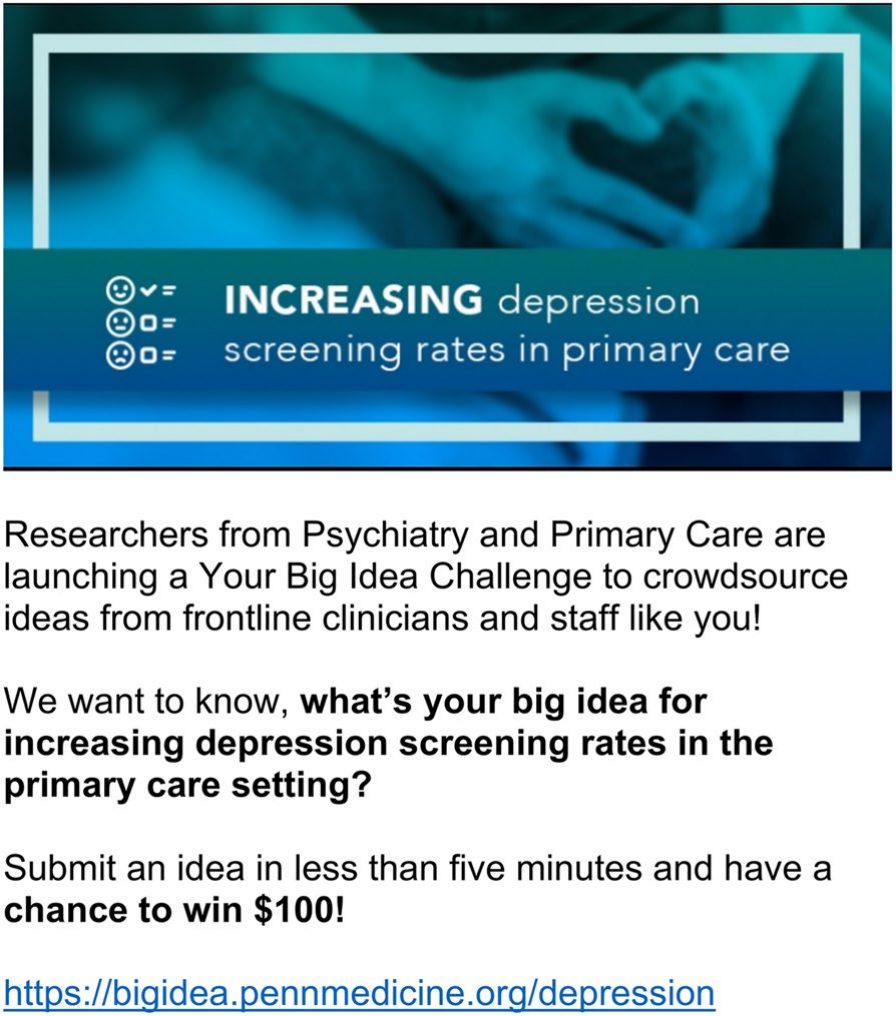
Flyer Advertising the Innovation Tournament in PIC practices

First, we invited all stakeholders (leaders from Primary Care and Psychiatry, clinicians, and staff) in PIC practices by e-mail to participate in the innovation tournament called “Increasing Depression Screening Rates in Primary Care.” Three emails in total were sent to a total of 420 participants. In terms of the tournament response, 150 stakeholders (36%) opened the first email and 17 (4%) clicked on the “Your Big Idea” link; 122 (29%) opened the second email and 15 (4%) clicked the link; 118 (28%) opened the third email and 14 (3%) clicked the link. To enhance participation, we posted flyers (see Fig. 1) advertising the innovation tournament at seven of the eight PIC primary care clinics; one program did not respond to the request. The team also spent time in staff rooms in three of the clinics to recruit clinicians and staff. The landing page for the innovation tournament, which was live between March 12 and April 5, 2019, is available for viewing at https://bigidea.pennmedicine.org/depression.

#### Results

##### Ideas coding

When the innovation tournament closed, 31 ideas and 32 barriers were submitted through the Your Big Idea platform from 12 primary care physicians, 10 MAs, 4 nurse practitioners, 2 practice managers, and 4 Patient Support Assistants (PSAs). The 31 ideas received 48 ratings; eight ideas received 5 stars (out of a 5-star system) and these top-rated ideas all proposed that patients complete the PHQ-2/9 via self-report either on tablet computers in the waiting area, by text message before their appointments, or through MyPennMedicine (Penn Medicine’s confidential online patient portal) before their appointments.

The research team organized a meeting with a panel of expert stakeholders and scientists to deliberate and vote on the innovation tournament winning idea. To reduce the burden on the panel, the research team refined the list of 31 ideas into themes using a content coding approach [53]. The team coded the 31 ideas together and when there were disagreements, they resolved them by consensus. The ideas were organized into four themes: (1) patient self-report (e.g., patients complete the screener on tablet computers in the waiting room, through MyPennMedicine communications, or by text message; *n* = 12); (2) reframing (e.g., changing the wording of the screener, changing the way it is introduced; *n* = 8); (3) workflow changes (e.g., putting screening results in the “Vitals” or “Chief Complaint” sections of the electronic health record Epic©, create reminders in Epic©; *n* = 7); (4) patient education (e.g., an anti-stigma campaign, clinician-provided psychoeducation about depression, flyers providing education regarding signs and symptoms placed strategically in clinics; *n* = 4). See Table 1 for the ideas, their respective themes, and the barriers identified by stakeholders.

**Table 1:** Themes, ideas, and barriers from the innovation tournament

Idea Theme	Number of Ideas	Idea	Barriers
Patient Education	4	“Make sure there is information concerning depression. Offer incentives for patients. Give some example of why people are depressed”	“Not knowing how to get help, not wanting help”
		“In addition to wraparound education on reducing stigma of depression and normalizing it as a mental health condition that can change over time, particularly with appropriate therapy/ resources, we could screen for depression using a “mood meter,” or some sort of “tool”, either a separate device or integrated onto screen, that gives brief explanation as to why we ask about mood state, then asks ‘over the last 2 weeks, I’ve been feeling “content / stressed / down/ depressed” etc. Each mood state gets its own color. If one selects, “depressed/down/hopeless” - the screen/ “meter” could light up a specific color, i.e., green or purple, and stay that color until the PCP arrives in the room. The PCP will see the very evident signal, and can start a conversation acknowledging mental health - even if to say, “it looks like the last 2 weeks have been going well for you! Great to hear.” Then move on with visit... obviously if light is “depression” signal, then PCP knows very clearly they need to follow up w/ [the PHQ]-9.”	“It gets overlooked because there is no standardized “checklist” or hard stop that ensures it gets done at every visit. There has been provider push back and gate keeping around when depression screening should be done, i.e. lack of recognition and prioritization despite evidence of benefit, and again no standardized expectation to perform and operationalize at the local level. We also need a greater focus on patient-centered efforts to explain why we are asking about depression on the delivery side as it may seem intrusive or confusing as to why we are seemingly “all of a sudden” more interested in if they’re depressed or not. I can imagine that this may feel suspect without a context.”
		“I believe the office should set a group/days aside in the office for depression/anxiety. So many people in the world are going through these issues alone.”	“Being afraid to speak up. That’s why with a group you can physically see and hear from real people that are suffering from the same issues. Also physicians do not have the time to access a depression visit and a sick visit.”
		“I don’t know if this idea will increase depression screening but I do think it has the potential to improve overall quality of care. I believe our current process, as it has been rolled out in my practice, is flawed. I believe it leads to inaccurate assessments and negatively impacts other aspects of the MAs rooming role including accurate BP [blood pressure] assessment. I believe this is a key aspect of what a provider should do with a patient, but we can think of prompts to avoid missing an opportunity when the agenda may overwhelm this issue. This could include simple signage that states something like – “Feeling blue? Talk to your provider.” “Feeling blue/sad? Please complete this form and talk to your provider.” The signs/form could be at the front desk, in the MA rooms or in the exam rooms. This is similar to a project I did several years go around smoking cessation that I think increased conversations but did not slow flow or lead to negative experiences for patients and our MAs.”	“The PHQ-9 is meant to be self administered. The MAs are ill-trained to do this effectively. Asking these questions while taking BP [blood pressure] negatively impacts both of these tasks. Depression needs to be considered at every visit and not based on some annual schedule. We are too wrapped up in process vs providing quality care.”
Reframe	8	“Change wording of questions & separate answers.”	“The way questions are being asked (feeling down, depressed or helpless). There’s no separation (they might feel down, but not depressed or helpless or vice or versa)”
		“Breaking down the questions in the PHQ-2. Change it to: “Have you been thinking about something that happened in your past recently that has been depressing?” This is because most people don’t relate it to as depression, but they are still thinking about things that make them sad.”	“The current wording of the PHQ-2 just doesn’t make sense to the patients. Patients will often say no to this question. But, if you explore more and ask them to talk more. The question is too long or the wording doesn’t make sense.”
		“I think that some of the questions that are currently on our depression screening can be confusing for the patient in order for them to respond appropriately. Clearer questions that are less intrusive on the surface could be beneficial.”	“Patients don’t always take these questions seriously and are just trying to get us out of the room in order to see their provider.”
		“How have you been feeling lately? Any feelings of being down or little interest?”	“People get really offended. I think it’s important for them to feel important and cared about. Some people take it that way or some people blow me off, like, [they say] “No,” with a face that you can almost feel like there is something they are not telling us. I sometimes feel like I want to say, “Are you sure? We have resources,” but I don’t want to be pushy if they say no. I think there should be more follow-up questions, something to make them feel like they need to speak up because it’s everyday they could be feeling like that and are almost hating life. I would hate for someone to not speak up and feel better.”
		“Maybe we shouldn’t limit the screening to just over the last 2 weeks. Depression isn’t always constant. Sometimes depression comes and goes, and 2 weeks is not a long enough time to determine that.”	“I’m not sure of any barriers but the procedure here at [XXX], the medical assistant goes over the health screening for every patient during triage. It covers fall risk, abuse, weight loss, advanced directives and depression screening. If they screen positive for depression we mention in the chief complaint “depression” and in the comments, we write “PHQ9” so once the physician takes over they will know to continue the depression screening.”
		“I would consider talking to the patient before asking the questions to see what the patient is going through in their life then ask the 2 questions pertaining to depression”	“The questions: Everyday, more than half the days and several days. Patient are sometimes confused about the days. The questions should be worded differently to accommodate the patients who are depressed but do not want to talk about it. After screening my patients, I ask about depression every visit because in my opinion I have a few that may slip through the cracks and the questions sometimes does not go across well, so I try to identify what’s going on with the patient an then ask the questions again if they are confused and state they are depressed; I ask them, if anything is going on in their life within the last 2 weeks that they may want to talk about that might cause them to have some kind of depression”
		“I think sometimes patients are reluctant to admit they may have depression, whether it is a cultural problem. Trying to make the patient feel comfortable during their visit so they admit there is an issue.”	“As above [barrier listed in idea]”
		“Get to know your patient, I think that all physicians should know their patients, and be able to identify if something major has changed in their lives, just by looking at them or noticing a big change in their weight, blood pressure or any life-changing events. My experience is when I go to the doctor, the assistants are not that approachable and I do not feel 100% comfortable talking to them about anything personal such as, depression, suicide, or abuse. My idea is for the physician to take 5 min to ask these questions themselves, so they can get a better understanding of what the patient is actually going through, I feel much better talking to my physician about suicidal thoughts, abuse from a loved one, or even depression, I don’t want no one going around looking at me different, I know there is HIPAA, but really who actually follows HIPAA when it’s someone you work with.”	“They seem reluctant to talk to the Medical Assistants, [patients would] rather speak to a nurse/or the doctor. There is not enough eye contact. The first question is too broad of a question? Do you have little interest or pleasure of doing things? Patients get a little taken. Patients think that some questions are weird, like ‘Do you have trouble dressing?’”
Self-report	12	“Each PSR holds a smart tablet and opens a bookmarked PHQ-2 screen and the patient logs into MPM [MyPennMedicine] during check-in to complete the 2 questions. If negative - a pop-up to let the patient know the result is negative and some evidence-based mental wellness practices like mindfulness-based stress reduction and regular exercise. If positive - a pop-up to inform the patient, recommend the patient tell the provider, offer general resources. If positive - a notification goes to the provider via HCM tab (or other location readily available to the provider) and telephone encounter. The patient hands the tablet back to the PSR, who resets the form (logs the patient out). Next patient checks in - rinse, wash, repeat. If the patient is not on MPM, then it’s a good opportunity to encourage signing up and the tablet could have a second MPM signup bookmark, which would increase the use of MPM.”	“The current state of our practice is to have MA’s provide the screen during check-in. This adds to MA workflow and can distract from other check-in needs.”
		“Patients could complete screening for depression on iPads while they wait to be called back for their appointment. This way it would be done for most patients before they even get into the room. Moderate or severe scores could trigger automatic referral to PIC to take the PCP out of the equation.”	“Time. Visits are limited and there is way too much to cover as it is.”
		“I think that we should just avoid a PHQ-2 altogether and hand the patient a PHQ-9 with check-in forms.”	“Sometimes the patient would like to explain why they feel the way they feel about every PHQ question which may take up time. We are not properly trained to handle certain scenarios when it comes to comforting the patient.”
		“Texting how you are feeling when you are feeling down or depressed to your primary care office or BH. Staff will respond back with a form screening questionnaire, based on answers, referrals are made to primary care provider, BH or crisis center is needed”	“Patients not answering questions honestly. (questions are asked by medical assistants and some patient may be embarrassed to really disclose how they are feeling)”
		“As a PSA for 24 years, I see over 140 patients in our practice daily. Sitting on the front line, I encountered some patients with depressive disorder, difficulty expressing how they feel on a daily basis, I try to comfort and support patients as much as I can. I inform them we care and want to help. I sometimes find myself tearing up listening to the stories they tell but at the same time, trying to stay calm and professional and to provide the assistance they may need. My idea would be to help our patients the best we can, to go above and beyond the call of duty. Being that the patients may not be comfortable, afraid to open up, we can create a short form that can be attached to the pre- visit summary. We print a pre-visit summary for each patient on a daily basis. This form can provide questions that may help us to get the best care. Patients can then give this form to our Medical Assistance to review. if patients are in urgent need of help, we can give the form to our CCBH worker.”	“Patients may feel uncomfortable talking about their issue at the front desk, patients dealing with something so difficult may have a hard time trusting someone not knowing how this person may react to their situation, its very scary to share something so personal. We have an open office filled with a great deal of patients daily. So it is a little uncomfortable opening up. [XXX] is our practice CCBH worker, [XXX] is our Social worker, they are very involved with our patients. Our patients feel comfortable with [XXX] and [XXX], these patients are escorted to a private area in the office to get the help needed. A short form attached to our pre-visit summary may be more comfortable and private for patients to document how they feel without someone knowing or hearing their current situation. This way, the patient can be pulled away from the front office to a secluded area in the office without anyone knowing. No patient should have to deal with this alone.”
		“I think that if the patients are allowed to answer the PHQ-2 questions on their own they would be more receptive when it comes to those questions”	“From my experience, I feel that the patients do not always answer the PHQ-2 questions honestly and I think that this is because there is such a frown upon depression.”
		“Patient could answer these questions in the privacy of their homes, workplaces, or settings. These questions can be populated into the patients’ chart prior to office visit, which makes the questions more accurate. The answers can be more accurate when answered by the patient. Patients are very hesitant to answer these questions when asked face to face. By offering this questionnaire in the online check-in process it will ensure completion.”	“Time is the biggest factor for screening patients for depression. By offering this feature, online check-in process cuts the time of triage by a great amount.”
		“Give patient access to Mychart either by iPad or computer while to enter answers to depression screening questions. This would also be a good way to ask about domestic violence, have patients check their med list and list their complaints in review of systems format etc. Offer a small incentive to patients for completing the screening questions such as a Starbucks gift card or a pen”	“Time and patient’s reluctance to answer questions with the medical assistant”
		“When I went to see a doctor at another institution 8 years ago, I was handed an iPad that was loaded by the check-in clerk with surveys and questionnaires that were selected based on the doctor I was seeing; my reason for visit; and my demographics including age and gender. By the time I saw the doctor, all that information was in the EHR and available for his use using a simple Smartphrase. The same occurs at CHOP when I visit a doctor with my child. Both sites use Epic. It is well established that patients are more likely to be forthcoming about personal issues with a tablet or kiosk than they are with a staff member (see, for example, https://www.annemergmed.com/article/S0196-0644(02)00080-X/abstract). My big idea is to make the provision of tablets a must-have element of EHR-based practice in every outpatient practice in the health system, and to have a PHQ-2 be part of the pre-visit survey completed on a tablet in the waiting room for patients due for screening.”	“The single greatest barrier to effective screening is the workflow that has the questions asked verbally by rushed and inexpert medical assistants, and recorded on paper for transcription by busy clinicians. There is no workflow that uses paper that works well in our practice, and this is a prime example. The other barrier is a philosophy that tablets are somehow a luxury for affluent (procedure-based, RVU-dense) practices, and not a must-have element of an EHR. Tablets should be as much a part of the IT outlay for an outpatient practices as keyboards, printers, and up to date computers. When we ask practices to pay for elements of the EHR out of revenues, it conveys a message that a complete EHR is only something you can afford if you do procedures. This leads providers to eschew the labor-intensive paper-based parts of the visit including depression screening.”
		“Send patient a MPM [MyPennMedicine] message 1-2 weeks before their appointment asking them to complete to save time at their appointment. This would allow them to complete in private without being rushed.”	“I worry that the screening done by MAs is so impersonal and rushed that patients don’t answer honestly, not unusual for patients coming in for depression to have 0/0 on PHQ-2.”
		“Send a pre-visit (within 24-48 h of the visit) message to patients via MPM [MyPennMedicine] enabled and showing no PHQ-2 screening in the previous year, similar to the plan for social determinants of health. They could complete the Smart forms before the visit and if positive could be flagged in the chart.”	“So far, I think our practice has really improved our screening of patients with the MA’s asking the PHQ-2 with vitals. However, there is a barrier that many patients are not being handed a PHQ-9 and, if they are, the PHQ-9 s are being scanned in instead of manually entered in PennChart [Penn Medicine’s version of Epic©] to satisfy having completed the measure. Certainly, it is a barrier asking this question to patients who have a long-standing history of depression/mood disorder. Those patients should either receive a PHQ-9 or have a documented discussion of the diagnosis in the visit, but we lack a workflow to identify those patients unless they are known to provider they will be seeing. Another barrier is time. When we screen for depression and it is a new finding, it is important and takes up a lot of our 20 min of time with the patient. We would love having more behavioral health staff on site for assistance with this workflow.”
		“Confidential survey for any and all patients to take prior to or during the wait for his/her provider. Survey will ask the same, if not more, detailed questions about whether or not they have felt depressed in the last 1-3 months. I’ve noticed that a lot of patients feel as though the 2-week screening does not help give their provider good insight on how they have been feeling overall. I feel this will also give the patient a sense of control and privacy. Some patients do not want to answer the questions when we, the medical assistants, ask because we are not “trained” or “do not have the skills and knowledge” to do so. Furthermore, I feel that this would give the patients who are unsure if they are experiencing depression or any other mental issue, a good idea of what signs and symptoms to look for in their daily lives...I would like them to be able to identify the difference between normal life stressors and extreme stressors that are prolonged, causing life disruptions.”	“Some patients do not want to answer the screening questions with anyone but the doctor because it is uncomfortable. Some patients state they do not need to answer the questions because they are receiving treatment elsewhere.”
Workflow changes	7	“I think it would be helpful for the medical assistants to be properly trained in asking the depression screening questions and how to properly respond to the patients who are currently going through depression.”	“A lot of the times the clinical staff is the first person to go over the depression screening with the patient and when a patient is feeling depressed and tries to discuss it with the clinical staff, I feel that the staff does not always know how to properly react toward that patient to comfort them or explain the process with the physician going over the next steps to help the patient. My fear is the patient tried to converse with the MA prior but the MA’s have such limited time with the patients when rooming and if the patient feels rushed or cut off by the time the physician comes in the patient may then feel closed off or shut the communication down since they couldn’t openly communicate with the Medical Assistant. I feel if there is a proper training for the clinical staff on communication with the patients for the depression screening both the patient and the medical assistant would feel more comfortable is asking the questions with a better understanding on how to react.”
		“At the [XXX] clinic, RN/MA’s are scoring PQH-2 during the check-in process. This is a perfect time to do screening as patients often are just waiting for their physicians to arrive. However, as a resident doctor I didn’t realize this was being done or where to look for the score until I asked another colleague in the clinic. It would be helpful to 1) do this screening in other clinics if not already adapted and 2) spread awareness that i) this screening is now in place at our practice and ii) where to look for the PHQ-2 score in the electronic system. This can be done by sending a bulletin email to the providers in the practice.”	“No significant barriers currently.”
		“I think the best way to increase screening is to actually remind the primary care provider to ask these two questions during the exam via a Best Practice Advisory flag once the chart is open and the patient is in the room. It should be a reminder only to complete the PHQ-2. If they want to, they can by-pass it but it may improve the number of patients screened if the primary care provider is aware that they do not need to directly address the results at the time of the appointment unless the appointment is for mental health purposes in which case the questionnaire is a moot point.”	“A provider believing that they will be forced to address the results at the current visit will create the perception that this screening tool is going to prolong the appointment and create further schedule delays.”
		“I have created a visit tool for the Medical Assistants to use in our office. The Medical Assistants use this form to remind them to review all of the meaningful use/quality questions. If the patient has a positive PHQ-2 the Medical Assistant documents this on the Chief Complaint along with the reason for the visit. Example. Patient here for follow up of hypertension (positive PHQ-2) the physician then knows to complete the PHQ 9.”	“The practice was understaffed for an extended amount of time.”
		“The idea would consist of asking all patients who are coming in for an annual exam or coming in with a set of chief complaints (insomnia, fatigue, sleep apnea, narcotic refill, back pain) the following question. “In the last 2 week have you noticed any change in your mood, be it worsening depression and anxiety”. If answered yes, this would prompt a PHQ2 or 9. One feature of this initiative would be the need for EPIC to capture this information as an adequate screen. This could simply be done by clicking a “yes/no” button that says. “I have screened this patient for depression.”	“There is often not sufficient time to do a full depression screen during a visit. Also, if depression is discovered, there is often not enough time to do a full psychosocial evaluation and discuss treatment. This often takes a full 30 min visit on it’s own and there are often other chronic health issues to discuss.”
		“The PHQ-2 screening is a good starting point. This can be done by medical assistants just as vital signs are done. The problem is finding the information in the chart and knowing if it’s even there to find. It’s unclear at each practice if PHQ-2’s are already being done all the time, some of the time, or never. Most primary care doctors include vital signs in their note. Personally, I review the vital signs that are unloaded into my note as I am writing the note and during the visit. If the PHQ-2 was added to the vital sign portion so it would be uploaded with typical vital signs or if more physicians included PHQ-2 results to upload into the note, I think this would increase depression screening. If the MA’s would give patients who screened positive on PHQ-2, the full PHQ-9 and GAD-7 questionnaires, this would save significant time and be very helpful for the visit.”	“Time, remembering to do it on all patients, not knowing if it was already done by the MA and where that information is”
		“Many barriers exist to screen adults for depression including the fact that many adults at risk for depression do not schedule or keep appointments for themselves. However, these adults may encounter primary care practices many times throughout the year when they present with family members including aging parents or young children. Pilot data across many primary care sites has demonstrated that screening mothers who attend well child visits is an effective platform to screen for depression and provide care for women at risk for depression. It is possible to build on this idea and extrapolate to other adults who present to primary care appointments with their spouses, partners or relatives. While not all family members will necessarily have their primary care within UPHS, a significant proportion will. Screening family members during routine primary care visits using a validated tool could close the gap in depression screening and improve care for patients and their families.”	“1. Limited time to address multitude of patient concerns 2. Workflow which does not effectively operationalize screening by non-providers 3. Absence of case management services within the practice”
	1	No idea	“Barriers: Not enough time during visits. Way too much to address at each visit; screening often takes a back seat to active issues. No quick way to identify last time someone was screened.”

*Note*. The innovation tournament submissions have been lightly edited for readability and blinded to protect participant confidentiality. One person did not submit an idea but did submit a barrier, which is listed in the bottom row of the table.

### Phase 2 — the panel of expert stakeholders & scientists

#### Methods

##### Participants

We invited a panel of expert stakeholders and scientists to discuss the ideas from the innovation tournament and to decide on the winning idea. To ensure that the panel was representative of all stakeholders involved in depression screening as part of the PIC program (from PSAs who check patients in at the front desk, to primary care clinicians, to patients, to social workers receiving referrals, etc.) we invited stakeholders from each group. To recruit MAs and PSAs, we asked the eight PIC practice managers to invite their staff to our upcoming meeting. To ensure patient participation, a primary care leader invited a patient from Penn Medicine’s primary care patient advisory board who had participated in the PIC program. In addition, two members of the research team—one expert in implementation science involved in the PIC program (CBW) and one expert in applying behavioral science methods to health systems (AMB)—were invited to participate. In total, the panel included six voting members: one social worker providing mental health services in the PIC program, one primary care physician in the PIC program, one leader in psychiatry, one patient from a PIC practice, and the two scientists on our research team. It should be noted that aside from one person, all other panelists had not participated in the innovation tournament and were therefore naïve to the tournament responses when discussing and rating them.

##### Procedure

The one-hour meeting took place over lunch. BSL summarized the innovation tournament results in the first five-minutes and guided the panel discussion with the following questions: “Which ideas are most surprising?”; “Are there any ideas you feel are missing from the list that would be important to test?”; “Which ideas can we immediately rule out?”; and “Which ideas seem most feasible, acceptable to clinicians and patients, and immediately actionable?”; and “Which ideas are your favorites?”

The panel discussed for 50 min and then anonymously rank-choice voted on their preferred idea theme (i.e., patient self-report, reframing, workflow changes, or patient education) using paper ballots. The discussion was transcribed verbatim.

The patient participating in the panel was compensated $100. Five innovation tournament participants with the most elaborated strategy to implement the winning idea were sent $100, and an additional five randomly selected innovation tournament participants were sent $100 to reward their participation.

##### Analysis plan

The discussion transcript was analyzed using content analysis to identify themes [53]. Two members of the research team (BSL and ACF) identified themes and repeating ideas. Disagreements were resolved by discussion and consensus. Rank-choice votes were analyzed using an instant runoff election method—a vote counting method for rank-choice elections— to select the winner of the innovation tournament [54]. The software used to analyze the election results was OpaVote©.

#### Results

##### Discussion themes

Table 2 displays the ideas and themes from the panel discussion. BSL and ACF identified several repeating ideas from the panel discussion. The discussion was divided into two parts: a discussion of the barriers to depression screening and a discussion of the ideas submitted to the innovation tournament.

**Table 2:** Themes and ideas from the panel discussion

**Barriers**	**Themes**	**Frequency**	**Representative Quotes**
Medical assistant administration of the PHQ-2	Medical assistant training is key	4	“Medical Assistants may not be appropriate to administer the PHQ, because they have very limited training. In other places, nurses do the screening and they’re much better trained, and the results are more accurate. It’s a much more costly option, but overall (not just for depression) it’s led to much better outcomes. Penn has decided to use medical assistants for vitals and you get what you pay for.”
			“Medical Assistants often have a great relationship with patients, and an interpersonal connection. I see the Medical Assistants in my practice stopping by patients’ doors and saying hello. They really have a deep connection. They could, with the right training, be important in getting the screenings done with the patients feeling comfortable.”
	The PHQ is not validated for clinician administration	4	“Self-directed PHQ-2 s are: (a) validated (it was how the tool was designed to be administered) and (b) gives the patient different options for how to fill it out (iPad, MyPennMedicine, etc.)”
Understanding the rationale for screening	Clinicians don’t understand	2	“The biggest problem is that many people don’t know what the concept of ‘screening’ is. It’s hard enough training residents on this, let alone medical assistants. For screening, you’re wanting to find the person who has slid under the radar, not the patient you already know has depression and is sad. That patient doesn’t need to be screened.”
	Patients don’t understand	2	“The patients are missing an explanation for why the practices are doing the screening in the first place and giving patients resources for what’s going to happen if they screen positive.”
Technological challenges	Health system technological challenges	2	“An idea that’s missing is that it is really hard to find the PHQ in PennChart [Penn Medicine’s version of Epic©] due to the way it’s configured. Doctors get very frustrated. Place it in a standard, permanent place in PennChart.”
			“In Psychiatry, no one knows where to find the PHQ-9 because they don’t have “vitals” on their dashboard. So, this presents problems.”
**Ideas**	**Themes**	**Frequency**	**Representative Quotes**
Reframing	Reframing is invalid	4	“Re-framing is the most surprising idea to me. I thought that we would see mostly self-report responses. The PHQ-2 is validated to be a self-report measure so it should be a self-report… Don’t change the items on the PHQ because it’s a validated measure.”
			“To me, the re-framing idea reflects the challenging piece that staff (medical assistants, residents, attendings) aren’t properly aware or trained about the PHQ-2 and aren’t fully knowledgeable about what screening is.”
Patient self-report	Tablet computers in the waiting area	5	“Do the PHQ-9 on tablets during waiting room downtime.”
			“If looking at patient screening as a long-term project, the percentage of people who are comfortable with technology will increase over time. So, it’s not a bad investment in the long-term.”
Patient education	Education is necessary	5	“Patient education is easy, quick, feasible to pilot. You can put signs in waiting rooms.”
			“One way to combine patient education and making this a workflow change, is potentially thinking about depression screening as the “fifth vital sign” like they did with pain.”

In terms of the barriers to screening, panelists were concerned about MAs administering the depression screener. The majority of panelists (67%) stated that MAs are not clinically trained to administer a sensitive mental health questionnaire. Most (67%) panelists voiced that the PHQ-2/9 were designed and validated for self-administration. Panelists (67%) also suggested that both clinicians, MAs, and patients may not fully understand the rationale for administering the depression screener. Finally, panelists (33%) indicated that there are several technological challenges related to the way depression screening results are integrated in Epic©.

Panelists discussed the ideas submitted to the innovation tournament. Most panelists (67%) focused on the fact that the “reframing” idea revealed that those submitting ideas to the innovation tournament were less focused on increasing screening rates, but rather improving the accuracy of screens. That is, tournament “reframing” ideas described ways to facilitate a deeper connection between providers and patients; they also recommended changing the wording of the PHQ-2/9 questions for the sake of clarity. The majority of panelists (83%) liked the idea of patient self-report of the depression screener, particularly the implementation of tablet computers in the patient waiting area. Panelists discussed safety and liability concerns with pre-check-in text messages through MyPennMedicine such as the need to ensure that the endorsement of suicidality could be quickly identified and acted upon if the patient is completing the PHQ-9 outside of the office. Most panelists (83%) also liked the idea of patient education and thought it was necessary, though one panelist voiced that patient education would unlikely increase rates; rather it would improve the accuracy of the depression assessment. Panelists felt that both ideas would also be feasible and acceptable to implement in the practices.

##### Election results

The instant runoff election winner to the rank-choice election was the patient self-report idea. The panel determined that the ideas suggesting patients complete the depression screener confidentially either on tablet computers, through MyPennMedicine ahead of the visit, or via text message were the most viable and potentially impactful strategies to improve depression screening.

### Phase 3 — piloting the winning innovation tournament strategy

#### Overview of rapid prototyping

Rapid prototyping is the systematic testing of ideas in order to create and refine strategies quickly [55]. First employed in industrial design, this method has been extended to healthcare contexts where effective implementation strategies don’t yet exist and immediate feedback is necessary to optimize healthcare quality and safety [38, 39, 48, 49, 56, 57]. Rapid prototyping facilitates learning as quickly as possible whether a strategy works and allows researchers to make adjustments as needed. Identified problems are documented and the implementation plan is revised. A subsequent experiment is conducted to see if it resolves the problem and to identify any further problems. Rapid prototyping is done iteratively and cyclically, much like Plan-Do-Study-Act cycles frequently used in quality improvement studies [58]. See the analysis plan below for the process description.

#### Method

##### Participants

To evaluate the feasibility and acceptability of the winning innovation tournament idea, we planned to first pilot in two PIC primary care practices before scaling the project up. The research team met with leadership in the Primary Care Service Line, including leaders from each PIC practice, and two practices agreed to participate in the pilot. Along with an informational technological consultant from the health system, two members of the research team met with the practice manager and the lead clinician of each practice to discuss the pilot workflow changes. Given that piloting the strategy would involve substantial changes to the workflow, one practice requested to initially pilot the tablets with one physician who already adhered to depression screening guidelines before scaling to the entire practice. Due to COVID-19 pandemic related physical distancing precautions, the pilot was halted after prototyping in one practice with one physician.

#### Procedure

##### Study design

Data collection was conducted according to a withdrawal design method—i.e., a method in which the intervention is “withdrawn” systematically to allow for a comparison between changes in the outcome in baseline versus intervention periods [59]. The research team was present on specific “intervention” days to use tablets for depression screening. On alternating “baseline” days, depression screening was conducted as it was normally conducted in the practice. In the case of this specific PIC practice, usual care for depression screening involves the MA verbally administering the PHQ-2, and (if indicated, i.e., a score of > 2) the patient self-administering the PHQ-9 using paper-and-pencil. Once the primary care clinician arrives in the examination office, the patient hands off the completed PHQ-9 for the clinician to manually enter in Epic©.

##### Materials

Two tablet computers were purchased to conduct the rapid prototyping. To ensure patient safety and confidentiality, the health system encrypted each tablet and installed Epic Welcome©, the patient-facing application version of Epic©. Patients complete questionnaires and consent forms on Epic Welcome© and their responses sync in real-time with the clinician-facing version of Epic©. On Epic Welcome©, when patients complete the PHQ-2, the questionnaire automatically expands into the PHQ-9 if patients’ score on the PHQ-2 is greater than or equal to 1 (endorsing at least some symptoms on either of the two items). Notably, this is a more liberal cut-off than CMS and health system guidelines, which recommend follow-up at a PHQ-2 score greater than 2. This discrepancy provides further evidence that significant technological barriers prevent health system standardization of depression screening, as described by innovation tournament participants and panelists.

#### Rapid prototyping analysis plan

Rapid prototyping was conducted over 5 days (i.e., five 5-h shifts) in a PIC practice. The three steps of the rapid prototyping process are outlined below.

##### Step 1 — design

Before each 5-h shift, members of the research team (BSL and ACF) designed a plan to use tablet computers to screen for depression. This plan included decisions about the workflow, materials needed, the staff involved in the process, and the location of screening. To ensure rapid feedback, the research team collected field notes and interviewed stakeholders (PSAs, the practice manager, MAs, the physician, and patients) involved in the pilot. Field note templates and qualitative interview guides are provided in Additional files 1 and 2 respectively. Qualitative interviews were transcribed verbatim in real-time using shorthand or on a laptop to ensure accuracy. The research team also documented whether the PHQ-2 was administered.

##### Step 2— evaluate and review

Immediately after a 5-h shift of rapid prototyping, the research team met to review the findings. Field notes and interviews were read aloud together and synthesized to eliminate redundancies and ascertain discrepancies. Qualitative data were analyzed using a rapid immersion/crystallization approach [36, 60–62]. BSL and ACF, who had been extensively immersed in the experience, developed impressionistic summaries of what they learned. Researchers’ holistic impressions of the experience were crystallized through discussion and written documentation. To ensure systematicity, the research team also recorded key features of each rapid prototyping cycle: (1) a summary of the workflow design; (2) screening results; (3) workflow successes; (4) workflow challenges; and (5) a summary of the changes to be tested in the next cycle.

##### Step 3 — refine and iterate

After determining the necessary workflow changes, the research team planned to refine the tablet screening process. This process sometimes involved writing scripts for the PSAs presenting tablets to patients to ensure that patients received uniform rationale about the PHQ-2. Other times, this involved placing laminated sheets with screenshots to guide MAs and the clinician to find the PHQ-2 depression screening data in their version of the electronic health record. The research team communicated with the practice ahead of the shift to ensure the changes were acceptable. The new iteration was then tested in the subsequent shift. In order to be able to directly evaluate whether the specific iteration of the strategy was superior to the previous cycle’s, attributes were not modified if they did not present challenges. This process repeated for each cycle.

#### Outcomes

##### Qualitative data

Field notes and interview transcripts from each cycle of the rapid prototyping process were collected and analyzed to iteratively improve the pilot process.

##### Quantitative data

The primary outcomes for the pilot were PHQ-2 and PHQ-9 screening rates and PHQ-2 follow-up. According to CMS in 2019, follow-up to the PHQ-2 (if the score is greater than 2) is considered complete if one of the following actions is taken: (1) the patient completes a more extended depression questionnaire (i.e., PHQ-9) or suicide assessment; or (2) the primary care clinician refers the patient to a mental health clinician; or (3) the clinician prescribes depression medications; or (4) the clinician documents a depression follow-up plan; or (5) the clinician documents a depression diagnosis.

Depression screening data were extracted from Epic©. Patient eligibility for the screener, PHQ-2 scores, PHQ-9 scores, medication list, medical diagnoses, referrals, and patient notes were extracted from Epic© for intervention and baseline shifts. To ensure a fair comparison and avoid any potential confounds related to the timing of the visit, baseline shift data were collected from the same time window and same physician as the intervention shifts. Because tablets automatically triggered the PHQ-9 at a lower score (a PHQ-2 score > 1) than CMS requirements and the usual care practice (a PHQ-2 score > 2), PHQ-9 and follow-up rates were compared statistically based on CMS requirements (PHQ-2 score > 2).

#### Results

##### Qualitative results

**Fig. 2:**
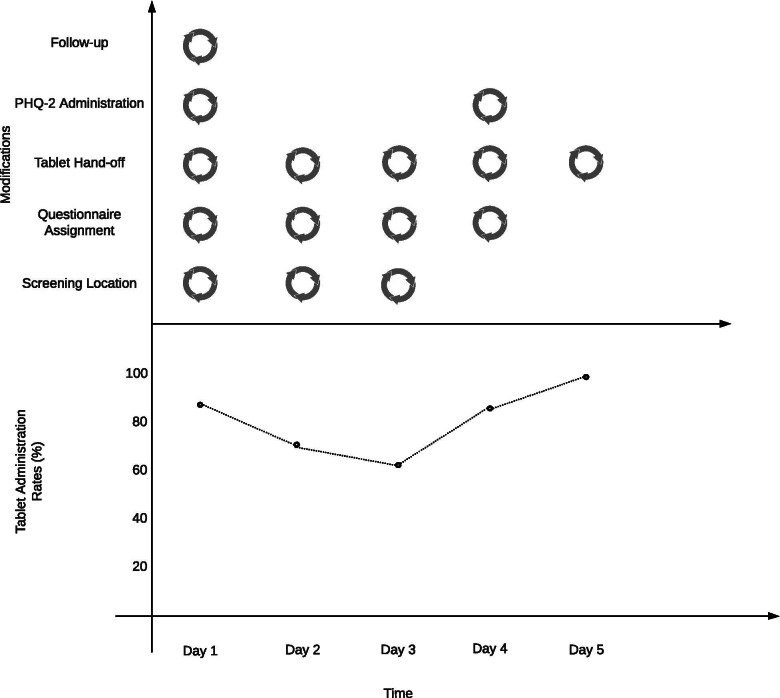
Rapid Prototyping Changes and Tablet Administration. Note. Rapid prototyping cycle icons are present in the upper window of the figure if modifications were made to this aspect of the piloting strategy based on decisions from the previous cycle. It should be noted that during piloting, though not all patients were administered the questionnaire via tablet, all patients completed the PHQ-2 (with the exception of one patient during the first cycle)

Table 3 displays the rapid prototyping process results from each day of piloting; Additional file 3 narratively describes these detailed results. Fig. 2 displays the modifications of each rapid prototyping cycle and tablet administration results.

**Table 3:** Rapid Prototyping Process for Tablets

		Day 1	Day 2	Day 3	Day 4	Day 5
**Design Summary**	**Screening Location**	Waiting Room	Exam Room	Waiting Room	Waiting Room	Waiting Room
	**Assigning the Questionnaire**	The Patient Service Associate (PSA) assigned the patient the questionnaire at check-in and provided the research team with the patient codes after check-in. The PSA would signal to research team when a patient needed to be screened.	The PSA assigned the questionnaire to patients at check-in. PSA would signal when a patient needed to be screened. Research team received the patient codes ahead of the shift and input the codes in the tablet.	The PSA assigned the questionnaire at check-in. PSA would signal when a patient needed to be screened. Research team received the patient codes ahead of the shift and input the codes in the tablet.	The PSA assigned the questionnaire to patients the morning before the shift (well before check-in). The PSAs entered the patient code in the tablets before handing the tablets to patients.	The PSA assigned the questionnaire at check-in. The PSAs entered the patient code in the tablets before handing the tablets to patients.
	**Tablet Hand-off**	Research team held onto tablets and approached patients with the tablet and took the tablet back from the patient.	Research team held onto the tablets. When the patient was called to the exam room we accompanied the patient and MA.	Research team held onto tablets and approached patients with the tablet and took the tablet back from the patient.	The PSAs gave patients the tablet at check-in. Patients were instructed to fill out the PHQ while in the waiting room and to bring back the tablet to the front desk as soon as they were finished.	The PSAs gave patients the tablet at check-in. Patients were instructed to fill out the PHQ while in the waiting room and to bring back the tablet to the front desk as soon as they were finished.
	**Administration Plan**	Research team assisted patients if they had questions.	Research team assisted patients if they had questions.	Research team assisted patients if they had questions.	Patient self-administered the PHQ-2.	Patient self-administered the PHQ-2.
	**Follow-Up Plan**	The plan was that if the patient screened positive, the Medical Assistant (MA) put down a red sheet of paper to notify physician.	The plan was that if the patient screened positive, the MA put down a red sheet of paper to notify physician.	The plan was that if the patient screened positive, the MA put down a red sheet of paper to notify physician.	The plan was that if the patient screened positive, the MA put down a red sheet of paper to notify physician.	The plan was that if the patient screened positive, the MA put down a red sheet of paper to notify physician
**Screening Results**	**Tablet Administration**	7 out of 8 patients completed the PHQ-2 on the tablet.	5 out of 7 patients completed the PHQ-2 on the tablet.	5 out of 8 patients completed the PHQ-2 on the tablet.	6 out of 7 patients completed the PHQ-2 on the tablet.	6 out of 6 patients completed the PHQ-2 on the tablet.
	**Verbal or Non-tablet Administration**	1 patient was roomed early and completed the PHQ verbally in the exam room with the MA.	1 patient completed the screener verbally because the PSA forgot to assign the questionnaire. 1 patient did it verbally because the patient was blind.	1 patient was very frail and elderly and could not operate the tablet and therefore completed the PHQ-2 verbally with the MA. 2 patients were called into the exam room before the research team was able to administer the questionnaire and completed the PHQ-2 verbally with the MA.	1 patient had completed the questionnaire on MyPennMedicine prior to their visit because the PSA had assigned the questionnaire before the appointment.	No non-tablet administration.
**Workflow Successes**	**Patient Perspective**	4 out of 7 patients indicated that they liked answering questions on tablets and that they were easy to use.	4 out of 5 patients indicated that the tablet was acceptable and more confidential.	5 out of 5 patients who used the tablet indicated that they were easy to use and that they preferred self-report over the MA asking them questions.	6 out of the 6 patients who used the tablet indicated that tablets were easy to use and fast. All of the patients brought the tablet back to the front desk when they were finished. Patients reported no barriers to completing the questionnaires on the tablets.	5 out of the 6 patients who used the tablet indicated that they liked it.
	**Staff Perspective**	PSAs said the process was fairly straightforward. They were initially confused about assigning the depression screening questionnaire (several processes and codes they needed to enter) but by the final patient, the time it took to assign the questionnaire decreased from as much as 5 min to less than 1 min.	The MA said that the process of completing the PHQ-2 in the exam room worked better than the waiting room because in the waiting room, patients don’t have privacy.	PSAs indicated that the process (assigning the questionnaire) was increasingly straightforward as long as they did not have to handle the tablets. They said the instructions were helpful and clear.	PSAs who had all participated in the pilot in previous shifts were now familiar with the workflow, with how to use the tablets, and how to introduce the study. PSAs also remembered to assign the questionnaire to all participants. The PSAs also handed the tablet to patients, so it was obvious which patients were the physician’s and who needed to be interviewed.	The MA indicated that the tablet process has been straightforward and cuts down on what they have to do, which is a benefit. They hoped that many other questions could be put on the tablet. PSAs described the final day of piloting as “smooth sailing” as they got accustomed to the process. They were surprised by how many patients remembered to return the tablets.
	**Clinician Perspective**	Physician did not report noticeable changes.	Physician indicated that from their perspective, things have been “working great.”	Physician did not report noticeable changes.	Physician did not report noticeable changes.	Physician indicated that from their perspective, the tablet process has been smooth.
**Workflow Challenges**	**Patient Perspective**	2 of 7 patients appeared to be confused by the tablets and/or questions and required assistance. 1 patient took a long time (20 min) to complete the PHQ.	1 patient who had completed the PHQ-2 on the tablet had not authorized their responses and therefore, the responses were not submitted. 1 patient was blind, suggesting a need for a back-up plan. 1 patient voiced strongly disliking the tablet. Patients took a long time to complete the tablet from 2 to 12 min. 1 patient asked that we use disinfectant wipes.	3 patients could not answer the depression screener on the tablet.	1 patient reported wanting an option to fill out the questionnaire online ahead of their appointment. Another patient expressed worries about getting sick from touching the tablets.	1 patient voiced extreme dislike of the tablets and said that they preferred to talk to a person.
	**Staff Perspective**	1 PSA indicated that it would be better to have the patient name, appointment. Time, and the CSN (identifying number) at the beginning of the rapid prototyping shift to assign before check-in.	PSAs expressed feeling overwhelmed, frustrated with the additional burden, and needing more explicit instructions and reminders to assign the patients the questionnaire.	PSAs expressed needing reminders to assign the questionnaires, because they sometimes forgot.	The 3 PSAs all expressed not liking the new workflow process, as it put more responsibility on their shoulders and was disruptive to the workflow. They said it was time-consuming and did not know what they would do if patients forgot to hand them back the tablets.	The MA expressed concerns about the scalability of the project. The MA indicated that for many elderly patients, tablets are infeasible, which means they would have to be screened by the MA anyway. The MA also said that if patients take long in the waiting area, this cuts down on the rooming time.
		1 PSA said that it would be nice to have a short script she could follow in order to inform patients when they arrive regarding the tablet pilot.	1 PSA reported not understanding the rationale for depression screening and feeling that it was unclear why screening was a priority.		1 of the PSAs had been proactive to avoid delays in check-in and assigned the questionnaires to the physician’s patients ahead of time (the morning of the shift). This inadvertently assigned the questionnaire through the confidential patient portal. One patient saw that the questionnaire had been assigned to them via email and completed the PHQ-2 prior to check-in.	The 2 PSAs indicated that the tablet screening required more time and disrupted the workflow. The PSAs preferred that the questionnaires be administered before check-in (online, through the confidential patient portal).
					Despite having disinfecting wipes, the PSAs reiterated concerns about illness (many sick patients were in the office).	
	**Clinician Perspective**	Physician had no constructive feedback this cycle.	The physician reported that the MA was placing the red paper down when the patient completed the PHQ-9 *not* when the patient scored positive on it. Physician said additional MA training was needed.	Physician indicated that despite additional training, the MA continued to place a red paper down even when the patient had not screened positive on the PHQ-9.	Physician had no constructive feedback this cycle.	Physician indicated that going forward suicide protocols were needed. In addition, physician felt it was important that the electronic health record had only place to enter PHQ data.
	**Technical and Workflow Challenges**	One of the tablets did not work.	The other tablet was still being fixed, leaving the team with only one tablet.	Research team did not have a patient list with names, making it difficult to tell which patients should be screened.	No technical or workflow challenges	No technical or workflow challenges
		Research team was stationed on the other side of the waiting room from the entrance and front desk. That made it difficult to identify patients for the questionnaire.	PSAs sometimes forgot to assign the questionnaire, and 1 PSA had not been trained in how to assign the questionnaire and, so, required additional training during a busy moment.	PSAs forgot to assign the questionnaires and had to be reminded.		
		Research team did not have the physician’s schedule ahead of time, so did not know which patients to look for or the code to enter in the tablet computer	In the exam room, the MA had to wait a long time for patients to complete the questionnaire, delaying the pre-visit vitals assessment. The MA still had to ask other questions to the patient and indicated that those questions should also be included in the tablet questionnaire.			
		The tablet only held a charge for ~ 3 h and needed to be plugged in.	Mid-rapid cycle prototyping shift, a new MA who had not been trained in the protocol saw the physician’s patients.			
**Summary of Changes to Test for Next Cycle**	**For Staff**	Create a 3-sentence script for PSAs to say when handing tablets to the patients.	Refine the PSA script and provide more specific instructions (with screenshots) for PSAs	Attempt to have PSAs hand off the tablet and ask patients to return the tablet to the PSA. Ask PSAs to assign all eligible patients the PHQ questionnaire before the shift to reduce time at check-in.	Provide additional disinfecting wipes to PSAs per their request (beyond the ones already given to patients).	No changes (last cycle).
			Retrain MAs about protocol and provide laminated instruction sheet. Ensure that the MA is only putting the red paper down when the patient screens positive, not just when they complete the PHQ-9.	Retrain MAs about protocol. Ensure that the MA is only putting the red paper down when the patient screens positive, not just when they complete the PHQ-9.		
	**Technical Changes**	Station ourselves next to the front desk and near an electrical outlet for ease of charge and for easier access to see patients and to hand the tablet to the PSAs.	One patient suggested disinfectant wipes to wipe down the tablet because many patients are sick. Research team will bring disinfecting wipes next time for the tablets.	No technical changes.	Revert to assigning the questionnaires at check-in to avoid patients completing the PHQ-2 at home through the confidential patient portal.	No technical changes (last cycle).
		Request the identifying number from the practice manager ahead of the shift so we can enter it in the tablet as soon as the patient checks in.	Research team will ask about the MA schedule to ensure that the MAs working during the next rapid prototyping cycle are trained.			
		Administer the patient questionnaire in the exam room.	Despite the privacy advantages of the exam room for the sake of time, administer the patient questionnaire in the waiting area.			
			Remind patients to authorize their responses when completing the PHQ-2 on the tablet.

Broadly, rapid prototyping revealed that tablet computers were acceptable and feasible to most stakeholders (the physician, the practice manager, MAs, and patients) involved in depression screening. However, PSAs found the additional responsibilities of checking patients in, handing off the tablets, and introducing the screener to patients to be somewhat disruptive to their workflow. In addition, anywhere between 1 and 3 patients during the first four five-hour shifts did not complete the questionnaire via tablet either due to the patient’s physical limitations, because the workflow was not yet mastered by stakeholders, or because there was not sufficient time to complete the depression screener via tablet. These patients completed the PHQ-2 verbally with the MA (i.e., through usual care). After several refinements were made during the rapid prototyping process, the process of depression screening via tablet improved and the PSAs eventually described that the workflow adjustments were “smooth sailing.” During the final shift, all patients were screened via tablet and stakeholders considered the pilot a “success.” Stakeholders made several recommendations for how to scale up the process across PennMedicine primary care practices, including transformations to Epic© and standardization of depression screening workflows across the health system.

##### Quantitative results

Overall, the rapid prototyping process resulted in comparable PHQ-2 screening rates to usual care (given that the practice participating in the pilot already screened all eligible patients). However, tablets significantly increased PHQ-9 screening rates. Follow-up between usual care and piloting days were comparable. *Z* test results for non-significant findings should be viewed in light of the fact that the pilot study was not adequately powered to detect a statistical difference.

On baseline days 30 out of 30 (100%) eligible patients were administered the PHQ-2. On intervention days 35 out of 36 (97%) of eligible patients were administered the PHQ-2. According to field notes, the patient whose PHQ-2 data were not recorded was given a tablet, indicating that responses were not synced with Epic©. Given that the practice participating in the pilot was highly compliant with PHQ-2 screening guidelines, differences between baseline and intervention days were not significant (*z* = 0.92, *p* = 0.36).

For those patients whose PHQ-2 score was positive (a score > 2), PHQ-9 screening was evaluated. On baseline days, 1 out of 3 (33%) of eligible patients were administered the PHQ-9. On intervention days, 6 out of 6 (100%) of eligible patients were administered the PHQ-9. Differences between baseline and intervention days were significant (*z* = − 2.27, *p* = 0.02).

Using the more inclusive definition of PHQ-2 follow-up per CMS guidelines (described above), follow-up rates were extracted from Epic©. On baseline days, 2 out of 3 patients (67%) received follow-up after a positive PHQ-2 screen. On intervention days, 6 out of 6 patients (100%) received follow-up after a positive PHQ-2 screen. Differences between baseline and intervention days were not significant (*z* = − 1.50, *p* = 0.13).

## Discussion

### Major findings

Our pilot study employed participatory and rapid implementation methods to increase universal depression screening in Penn Medicine primary care practices. First, we employed an innovation tournament to gather ideas from stakeholders (leaders from Primary Care and Psychiatry, clinicians, and staff) about how to increase depression screening in primary care. Second, a panel of expert stakeholders and scientists deliberated and voted on the best innovation tournament idea to pilot. The panel determined that rather than the usual care practice of verbal PHQ-2 administration by MAs, the research team should pilot an electronic self-report method. Third, we piloted this winning idea in one primary care practice with one physician over 5 cycles. Using an innovative rapid implementation method called rapid prototyping, we designed and refined a strategy to screen patients for depression with tablet computers. Our pilot study found that using a tablet for patient self-administration of depression questionnaires was feasible and acceptable across stakeholder groups, though PSAs expressed concern about the additional responsibilities resulting from these workflow changes. Despite significant workflow changes, in our limited sample PHQ-2 screening rates using tablets were comparable to usual care screening rates given that the practice/physician we worked with already accomplished universal (i.e., 100%) screening rates. Moreover, PHQ-9 screening rates were significantly higher using the tablet. Follow-up rates for usual care and intervention days were comparable. PHQ-9 screening rates were likely higher because, unlike usual care at the practice that tasked MAs with following up on the PHQ-2, tablet computers automatically triggered and generated the PHQ-9 screener if a patient screened positive on the PHQ-2. Whereas usual care necessitated continuous and deliberate human intervention, the app on the tablet computer generated the PHQ-9 by default.

### Strengths and weaknesses of participatory and rapid implementation approaches

This case study revealed the advantages and disadvantages of using participatory and rapid implementation approaches to strategy design. This study is unique for its inclusion of a variety of stakeholder perspectives across the three study phases. As a consequence of health system incentives for physicians to see more patients and meet more demands, more responsibilities—formerly within the sole purview of physicians—are being shifted onto non-physician and often non-clinical workers [63]. The few studies that have sought the perspectives of non-physicians have discovered that health system transformations rarely incorporate their often diverging concerns [64, 65].

Consistent with this work, the panel discussion of expert stakeholders and scientists revealed that different stakeholder groups have different knowledge and priorities about screening that need to be addressed by health systems [66–68]. For example, the panelists felt that the reframing ideas by the diverse innovation tournament participants showcased a misunderstanding about the rationale for screening. The panelists also discussed some of the drawbacks of task-shifting through the employment of non-clinical staff and MAs for screening. The panel suggested that clinicians, staff, and patients may require additional education to better understand the rationale for depression screening. They also discussed that not all clinicians feel equipped or comfortable with providing depression follow-up. These factors influence the accuracy of the depression screen and may indirectly impact screening rates [66, 69–73]. Field notes and qualitative interviews from the rapid prototyping phase again displayed conflicting stakeholder perspectives. Though most patients found depression screening on the tablet acceptable, a contingent could or would not use the tablets for various reasons (e.g., physical ability, digital literacy, concerns about the impersonality of the questionnaire). PSAs felt that they were being given more responsibilities that were disruptive to the workflow and one expressed not understanding why depression screening was a priority, whereas clinical staff found the tablet screening acceptable and feasible.

These diverging stakeholder views may reflect differences in responsibilities, interest, knowledge, and stigma related to mental health issues. Health systems must incorporate and address these different perspectives in future implementation efforts to ensure their success. For example, future efforts using tablets to screen for depression might consider developing a back-up plan for patients who dislike or cannot use a self-report electronic screener and consider reducing PSA burden. In addition, though the clinical staff in the rapid prototyping phase were eager to expand these strategies to other mental health conditions and to standardize the physician-administered suicide assessment protocol, based on our prior work in the PIC program, some clinicians prefer to hand off psychiatric and suicide assessments to the PIC mental health provider [40]. Implementation strategies for depression follow-up, including assessing suicide risk, that can assist staff and clinicians with varying levels of comfort treating psychiatric conditions may be needed as health systems integrate mental and physical health services.

Another strength of the rapid implementation approach was the ability to get feedback quickly from stakeholders who were invested in the quality improvement effort. The innovation tournament was an efficient method to reach engaged stakeholders in the PIC program and the panel meeting enabled the research team to promptly evaluate the feasibility of the innovation tournament ideas. Rapid prototyping allowed the research team to receive immediate feedback on whether the stakeholder-proposed changes were acceptable, feasible, and increased screening rates. In our pilot study, several of the stakeholder-proposed changes were successful, and others were not (e.g., conducting the PHQ-2 in the exam room). We were able to present the data to stakeholders and rapidly change course when there was agreement that the current iteration of the strategy was ineffective. Altogether, our work suggests that there are significant advantages for researchers to employ these methods. Rapid experimentation that engages the entire workforce involved in health system transformations is the necessary next step to realize the promise of a learning health system [39, 56].

There were also several weaknesses to these participatory and rapid implementation methods. Most significantly, the stakeholders engaged in the process (from the innovation tournament to the rapid prototyping phase) were heavily invested in system change, concerned about increasing depression screening, and motivated to voice their input in the research process. These stakeholders were not representative. The stakeholders that chose to participate in the tournament tended to provide highly elaborate responses, which accords with the literature suggesting that the most engaged stakeholders who are eager to share their ideas will submit ideas to the tournament [46, 47, 51, 74].

Stakeholder self-selection was perhaps most evident in the high practice screening rates at baseline. We worked with a practice/physician for the pilot that already had high PHQ-2 screening rates so the practice felt assured they would not risk compromising patient care during the pilot. However, similar to other health systems, depression screening rates across Penn Medicine primary care practices are far more variable, ranging from 10 to 90% [69, 72, 75, 76]. The level of engagement and motivation in the process is not surprising given our approach, which relies on innovators (i.e., those interested in and willing to engage in health system experimentation). These methods are likely best suited for the early stages of innovation diffusion [77]. Though the innovation tournament fielded ideas from stakeholders across PIC practices, it is important to test whether the tablet strategy will generalize to other primary care practices where depression screening and stakeholder investment may be lower. It is possible that at other practices where there is less buy-in or motivation, social incentives and peer comparison interventions would be needed to complement these efforts [78, 79]. Going forward, leveraging the leadership and embeddedness of implementation champions will be crucial to effecting large-scale change in the entire health system [80].

### Study limitations and future directions

Beyond the limitations of the methods, our study also has several specific limitations. For one, the results of the innovation tournament were heavily influenced by the framing of the Big Idea prompt. The research team intended for respondents to generate innovative ways to increase PHQ-2/PHQ-9 use, yet many respondents focused on ways to improve depression screening that did not involve these specific measures. Though we learned much about stakeholders’ attitudes and knowledge about depression screening from the innovative tournament results, which informed our investigation, in future research we would likely focus the Big Idea prompt on the PHQ-2/PHQ-9 to generate more targeted responses. A related limitation was the absence of patient participants in the innovation tournament. Given how much we learned about diverging stakeholder perspectives throughout the pilot study, it is important to understand how patients would prefer to be screened especially because verbal administration of the PHQ-2 (the current screening protocol at many of the primary care practices) is not psychometrically valid. Moreover, patient-centered depression care is associated with improved treatment outcomes [81].

Third, we were not able to scale up the pilot beyond one physician in one practice due to the onset of the COVID-19 pandemic, which halted research. Though our study yielded important lessons on innovative participatory and rapid methods to improve implementation design, depressing screening rates from the pilot study should be appropriately contextualized in light of the small sample. Researchers are planning to conduct further examinations in the health system to scale up this experimentation when physical distancing guidelines are no longer in place. One potential benefit from the COVID-19 pandemic was the rapid switch to telehealth, during which health systems became well-versed in virtual mental health screening and risk assessment. Health systems may now be less hesitant to screen patients ahead of their primary care visits through confidential patient portals or via other methods due to liability issues regarding the endorsement of suicidality on the PHQ-9. Increasing the availability of options for patients to complete the PHQ-2 and PHQ-9, including ahead of their primary care visit, would likely improve depression screening rates and should be explored by health systems.

Another limitation of our study is that due to the preliminary nature of the investigation, permanent modifications to the electronic health record that would have affected the whole health system were not feasible. For example, through Epic© it is possible to automate the assignment of the depression questionnaire to avoid PSA burden. Yet due to the preliminary nature of our study, the research team was not able to execute this change. During the rapid prototyping process, PSAs found assigning the PHQ-2 to be disruptive and burdensome. This particular challenge may not generalize to a large-scale implementation initiative where such changes would be permitted.

## Conclusions

Employing participatory and rapid implementation methods to increase depression screening in primary care is effective at engaging stakeholders, generating investment in the project, and improving the design of implementation strategies. Our findings reveal that involving all stakeholders impacted by these implementation efforts can provide important guidance for how to effect large-scale change in the health system. Health systems and payers must attend to the diversity of perspectives from all stakeholders affected by transformations in healthcare provision. In particular, while electronic depression screening is considered a priority for many stakeholders, our pilot study found that a contingent of patients could not complete this method and that PSAs were saddled with additional responsibilities that they perceived to be disruptive. To accurately capture depression rates, screening practices must allow for implementation flexibility. More broadly, our preliminary results suggest that these methods can improve universal depression screening in primary care practices. We were able to leverage participatory and rapid approaches to design implementation strategies to improve screening at relatively low cost, with sustained stakeholder engagement and buy-in, and without disrupting workflows permanently. Health systems committed to implementing evidence-based practices beyond depression screening stand to gain from these rapid, stakeholder-centered design methods.

## Supplementary Information

**Additional file 1.** Field Notes Template.

**Additional file 2.** Rapid Qualitative Interview Template.

**Additional file 3.** Rapid Prototyping Results.

## Data Availability

The datasets generated and analyzed during the current study are not publicly available due to the sensitive and identifiable nature of qualitative data and small pilot study data, but de-identified data are available from the corresponding author on reasonable request.

## Abbreviations

PIC: Penn Integrated Care
MA: Medical Assistant
PSA: Patient Support Assistant
PHQ: Patient Health Questionnaire
CMS: The Centers for Medicare & Medicaid Services
