# Supplementary material for: A pilot study of participatory and rapid implementation approaches to increase depression screening in primary care
Source: BMC Fam Pract. 2021 Nov 16;22:228. doi: 10.1186/s12875-021-01550-5 (PMC8593851; doi:10.1186/s12875-021-01550-5)
Supplement: Supplementary file 1 — Additional file 1. Field Notes Template. [file 12875_2021_1550_MOESM1_ESM.docx]

**Field Notes Template**

**General Practice Observations**

**Practice ID:**

**Date:**

**Time:**

**Observations about the Waiting Room**

**Approximately, how many patients are in the room?**

| **0 hour:** | **2 hours:** | **4 hours:** |
| --- | --- | --- |
| **0.5 hour:** | **2.5 hours:** | **4.5 hours:** |
| **1 hour:** | **3 hours:** | **5 hours:** |
| **1.5 hours:** | **3.5 hours:** | **5.5 hours:** |

**What are patients doing in the waiting room?**

| **P1:** | **P2:** | **P3:** | **P4:** |
| --- | --- | --- | --- |
| **P5:** | **P6:** | **P7:** | **P8:** |

**How long is the patient in the waiting room?**

| **P1:**  **Appt Time:**  **Arrival:**  **Called In:** | **P2:**  **Appt Time:**  **Arrival:**  **Called In:** | **P3:**  **Appt Time:**  **Arrival:**  **Called In:** | **P4:**  **Appt Time:**  **Arrival:**  **Called In:** |
| --- | --- | --- | --- |
| **P5:**  **Appt Time:**  **Arrival:**  **Called In:** | **P6:**  **Appt Time:**  **Arrival:**  **Called In:** | **P7:**  **Appt Time:**  **Arrival:**  **Called In:** | **P8:**  **Appt Time:**  **Arrival:**  **Called In:** |

**What are the staff doing?**

**Are there any other notable observations about patients, staff, providers, or the setting? General climate?**

**Tablet Observations**

**How many patients does the provider have scheduled for this time window?**

**Observations of the PSA when handing the patient the tablet (body language, statements made, etc.).**

**How long does it take the PSA to assign the patient the questionnaire?**

| **P1:** | **P2:** | **P3:** | **P4:** |
| --- | --- | --- | --- |
| **P5:** | **P6:** | **P7:** | **P8:** |

**What is the patient’s initial reaction when they are handed the tablet (body language, statements made, etc.)?**

| **P1:** | **P2:** | **P3:** | **P4:** |
| --- | --- | --- | --- |
| **P5:** | **P6:** | **P7:** | **P8:** |

**Observations of the patient as they complete the PHQ (body language, statements made, any difficulties noted).**

| **P1:** | **P2:** | **P3:** | **P4:** |
| --- | --- | --- | --- |
| **P5:** | **P6:** | **P7:** | **P8:** |

**How long did it take the patients to complete the questionnaire?**

| **P1:** | **P2:** | **P3:** | **P4:** |
| --- | --- | --- | --- |
| **P5:** | **P6:** | **P7:** | **P8:** |

**Were there any noticeable barriers?**

| **P1:** | **P2:** | **P3:** | **P4:** |
| --- | --- | --- | --- |
| **P5:** | **P6:** | **P7:** | **P8:** |

**Any other noted observations.**
