# Supplementary material for: A pilot study of participatory and rapid implementation approaches to increase depression screening in primary care
Source: BMC Fam Pract. 2021 Nov 16;22:228. doi: 10.1186/s12875-021-01550-5 (PMC8593851; doi:10.1186/s12875-021-01550-5)
Supplement: Supplementary file 2 — Additional file 2. Rapid Qualitative Interview Template. [file 12875_2021_1550_MOESM2_ESM.docx]

**Rapid Qualitative Interview Template**

**Practice ID:**

**Date:**

**Time:**

**For providers, front-desk staff, MAs, nurses:**

1. **Tell me about your experience with the tablet screening for depression.**
   1. [Probes] Did you notice the change?
   2. [Probes] Did it get in your way?
   3. [Probes] Did it make anything easier? Do you have any suggestions to make it better?
2. **What are things you like about using the tablets for depression screening?**
   1. [Probes] Do you welcome the change? Is it appealing? Do you approve?
   2. [Probes] Are the tablets/workflow easy to use and understand?
3. **We tried this process with just a small number of physicians and medical assistants. Do you think we could use this process with the whole practice?**
   1. [Probes]: What do you think makes it possible, doable, and implementable?
4. **What are things you dislike about using the tablet for depression screening?**
   1. [Probes] What are some of the challenges to using the tablet?
   2. [Probes] What are the barriers to making tablet screening possible, doable, and implementable?
5. **How would you make this process better? Other suggestions for how to increase depression screening?**

**For patients:**

1. **What was it like to use the tablet to answer questions about your well-being?**
2. **What are some things you liked about using the tablets?**
3. **What are some things you didn’t like?**
4. **Have you ever been asked these questions about your well-being before? How were you asked? Which method do you prefer? How would you prefer to be asked these questions?**
5. **Do you trust your doctor?**
   1. Scale from 0 to 10, with 0 being you don’t trust your doctor at all and 10 being that you that feel you can tell your physician anything and that you trust his/her judgment with regard to your care?
6. **Is there anything you can think of that would make screening for your well-being better?**
