# Supplementary material for: A pilot study of participatory and rapid implementation approaches to increase depression screening in primary care
Source: BMC Fam Pract. 2021 Nov 16;22:228. doi: 10.1186/s12875-021-01550-5 (PMC8593851; doi:10.1186/s12875-021-01550-5)
Supplement: Supplementary file 3 — Additional file 3. Rapid Prototyping Results. [file 12875_2021_1550_MOESM3_ESM.docx]

**Rapid Prototyping Results**

On the first day of piloting the tablets, BSL and ACF screened patients in the waiting room. The PSAs assigned patients the PHQ-2 and provided the research team with a specific patient code that the team needed to input in the tablet to connect patients’ PHQ-2 responses to Epic©. The PSA would signal to the research team to approach the patient. The research team assisted patients as they completed the questionnaire and 7 of the 8 patients (88%) completed the PHQ-2 on the tablet, with one who didn’t because they were called into the exam room before there was an opportunity for tablet screening. Most (57%) of the 7 patients found the tablets easy to use and acceptable; the remainder (43%) found the tablets confusing or took a significant amount of time to complete the questionnaire. PSAs indicated that the workflow was relatively straightforward, but they requested scripts so they could know how to approach patients about the study. BSL and ACF also worked collaboratively with the PSAs to create a frictionless workflow to ensure that patients could complete the questionnaire immediately after check-in. PSAs planned to give the research team the patients’ questionnaire codes ahead of the shift so that they could enter the code as soon as patients were checked in. The PSAs and the MA wondered whether it was possible to administer the PHQ-2 in the exam room, and the research team resolved to try this method of administration in the next rapid prototyping cycle.

On the second day, the research team made changes from the first cycle. BSL and ACF administered the PHQ-2 in the exam room. They received patients’ codes ahead of the shift and were able to input the codes themselves when the PSA signaled a patient had arrived for their appointment. When the patient was called into the exam room by the MA, the research team accompanied the patient to complete the questionnaire, which proved challenging. Patients took a long time to complete the screener (*M* = 5.6 minutes), which delayed the MA from assessing patients’ vitals. Five of 7 patients (71%) completed the screener by tablet, with one who did it verbally because a PSA forgot to assign the questionnaire and another because they were blind. Most (80%) of patients found the tablets easy to use and acceptable, indicating that the tablets were more confidential. The one patient who did not like the tablet reported concerns about their cleanliness and indicated that they preferred human interaction: “I don’t like computers. I prefer to talk to someone. Whenever I use a computer, I feel like it’s taking someone’s job.” The MA indicated that they preferred patients completing the screener in the exam room because they had more privacy. The PSAs expressed feeling overwhelmed with the additional burden of the tablet screening process with one PSA indicating that the rationale for depression screening was not clear or adequately explained. They requested additional reminders for assigning the questionnaire and more explicit instructions for patients. The physician indicated that the workflow was going well, but that the MA was placing a red piece of paper down whenever patients completed the PHQ-9, not when the patient scored positive on it as instructed, indicating that additional MA training was needed. Other minor challenges arose in the rapid cycle prototyping process including the PSAs sometimes forgetting to assign questionnaires and a personnel switch mid-shift in which a new MA took over responsibilities who was not aware of the study protocol. In response to this cycle, the research team planned to refine the script for PSAs and provide more specific instructions for them (with screenshots) on how to assign the PHQ-2 to patients. The research team also planned to retrain the MAs about the protocol and provide laminated instruction sheets to ensure that the MA would only put the red paper down when the patient’s score on the PHQ-9 merits follow-up. The research team also bought disinfectant wipes for the tablets. BSL and ACF determined that due to time constraints, administering the PHQ-2 in the waiting area was preferable.

On the third day, the research team made changes in response to the second cycle. BSL and ACF administered the PHQ-2 in the waiting area. As in the last cycle, they received patients’ codes ahead of the shift and were able to input the codes themselves when the PSA signaled a patient had arrived for their appointment. The research team assisted patients with PHQ-2 administration. Five out of 8 patients (63%) completed the questionnaire by tablet, with 1 patient who was too frail to operate the tablet and 2 patients who were called into the exam room before the research team was able to administer the PHQ-2. Of the patients who were able to use the tablet, all 5 (100%) indicated that the tablet was easy to use and that they preferred self-report over MA-administration. Problems from the second cycle were not fully resolved in terms of improving MA and PSA workflows. As such, the research team committed to meeting with the MAs again to troubleshoot and to give the PSAs the tablet to avoid the research team as middleman.

On the fourth day, the research team made changes from the third cycle. One PSA assigned patients the PHQ-2 the morning before the shift in order to prepare for the rapid prototyping cycle. When patients checked in, the PSAs entered the patient codes in the tablets and handed them to patients with specific instructions to bring them back when the patient completed the questionnaire. Six of the 7 patients (86%) self-administered the PHQ-2, with one patient who had already completed the questionnaire prior to their visit via the confidential patient portal, MyPennMedicine. From this encounter, the research team learned that the PSA’s assignment that morning had triggered emails to patients to fill out the depression screener on MyPennMedicine. All 6 (100%) of the patients who used the tablets indicated they were easy to use and quick. One patient indicated wanting an option to complete the questionnaire ahead of their appointment at home, and another expressed concerns about getting sick after touching the tablet. All patients brought the tablet back to the front desk when they were done without prompting. All 3 PSAs expressed concerns about the new workflow process, as it tasked them with more responsibilities and was disruptive and time-consuming. They wondered what they would do if patients forgot to hand them back the tablets. They also asked for additional disinfectant wipes. The research team decided to caution PSAs against assigning the questionnaires ahead of time given the legal department’s concerns about screening patients before visits.

On the fifth and last day, the research team made the planned changes from the fourth cycle. The PSAs assigned the questionnaires at check-in, entered patients’ codes, and handed patients the tablets with explicit instructions to return them upon completion. All six (100%) patients self-administered the PHQ-2, with 5 of the 6 (83%) indicating that they liked tablets and found them easy to use and one patient expressing dislike for tablets and preferring in-person communication. The MA indicated that the tablet process was straightforward and reduced their workload. They hoped that many other questions could be put on the tablet. PSAs described the final day of piloting as “smooth sailing” as they were finally accustomed to the process. The PSAs expressed surprise that patients remembered to return the tablets. The PSAs continued to emphasize, however, that tablet screening required additional time and disrupted the patient check-in workflow. The PSAs preferred that the questionnaires be administered before check-in (online, through the confidential patient portal). In terms of workflow challenges, the MA expressed concerns about the scalability of the project. The MA indicated that for many elderly patients, tablets are infeasible, which means they would have to be screened by the MA anyway. The MA also said that if patients take a long time in the waiting area with the tablets, this could potentially cut down on the rooming time. The physician considered the pilot study a success and expressed hope that going forward, a robust suicide protocol could be put in place. In addition, the physician felt it was crucial that transformations to Epic© would systematize where depression screening data are entered and retrieved on the electronic interface.
